# Evidence of Coinfections between SARS-CoV-2 and Select Arboviruses in Guerrero, Mexico, 2020–2021

**DOI:** 10.4269/ajtmh.21-1216

**Published:** 2022-01-24

**Authors:** Daniel Nunez-Avellaneda, Fabian R. Villagómez, Julio C. Villegas-Pineda, Jacqueline Barrios-Palacios, Ma. Isabel Salazar, Carlos Machain-Williams, Bradley J. Blitvich

**Affiliations:** ^1^ College of Veterinary Medicine, Iowa State University, Ames, Iowa;; ^2^ Laboratorio de Microbiología Molecular, Escuela Nacional de Ciencias Biológicas del Instituto Politécnico Nacional, Ciudad de México, Mexico;; ^3^ Departamento de Microbiología y Patología, Centro Universitario de Ciencias de la Salud, Universidad de Guadalajara, Guadalajara, Jalisco, México;; ^4^ National Institute of Medical Sciences and Nutrition Salvador Zubirán, Experimental Pathology Section, Ciudad de México, Mexico;; ^5^ Laboratorio de Virología e Inmunovirología, Depto. Microbiología Escuela Nacional de Ciencias Biológicas Instituto Politécnico Nacional, Ciudad de Mexico, Mexico;; ^6^ Laboratorio de Arbovirologia, Centro de Investigaciones Regionales “Dr. Hideyo Noguchi,” Universidad Autonoma de Yucatan, Merida, Yucatan, Mexico

## Abstract

We provide evidence of concurrent and close sequential infections between SARS-CoV-2 and select arboviruses—namely, chikungunya virus (CHIKV); dengue viruses 1, 2, and 3 (DENV1–3), and Zika virus (ZIKV)—in patients in Guerrero, southwest Mexico, in 2020–2021. The study population consisted of 176 febrile patients with laboratory evidence of SARS-CoV-2 infection. Sera from all patients were serologically and antigenically tested for seven arboviruses known to occur in Guerrero. Eighteen patients contained CHIKV IgM, six of whom also contained CHIKV RNA. Another 16 patients contained flavivirus antigen. The flaviviruses responsible for the infections were identified by plaque reduction neutralization test as DENV1 (two patients), DENV2 (five patients), DENV3 (three patients), ZIKV (three patients), and an undetermined flavivirus (three patients). In summary, we identified patients in Guerrero, Mexico, with concurrent or recent sequential infections between SARS-CoV-2 and select arboviruses, exemplifying the importance of performing differential diagnosis in regions where these viruses cocirculate.

Severe acute respiratory syndrome coronavirus 2 (SARS-CoV-2) is the etiological agent of COVID-19, which is characterized by various clinical manifestations, including acute undifferentiated febrile illness, in humans.^1^ Others causes of acute undifferentiated febrile illness in humans include chikungunya virus (CHIKV), dengue viruses 1 to 4 (DENV1–4), West Nile (WNV), and Zika virus (ZIKV), all of which are arthropod-borne viruses (arboviruses) associated with fatal disease outcomes.^2^ CHIKV, DENV1-4, WNV, and ZIKV occur in Mexico and elsewhere in Latin America, complicating the diagnosis of COVID-19 in this region. The goal of this study was to assay febrile patients in Guerrero, a coastal state in southwest Mexico, for concurrent SARS-CoV-2 and arbovirus infections.

The study population consisted of 176 patients from Guerrero who had laboratory confirmed acute SARS-CoV-2 infections, probable acute or recent SARS-CoV-2 infections, or probable past SARS-CoV-2 infections. The patients presented with acute undifferentiated febrile illness in June 2020 to March 2021 at three participating sites in Guerrero: the Hospital General Adolfo Prieto in Taxco de Alarcón (HGAPTA), Laboratorio de Análisis Clínicos Avellaneda in Chilpancingo (LACAC), and Labymedic Laboratorios in Acapulco (LLA). As noted earlier, all patients presented with unspecified febrile illness, but the medical personnel at the participating performance sites were not willing to provide any other clinical information because of the time needed to compile these data. Nasopharyngeal swabs were collected from select patients and serum samples were collected from all patients. If a swab was collected, the patient was tested for SARS-CoV-2 RNA by quantitative reverse transcriptase polymerase chain reaction (qRT-PCR) using the DeCoV19 Kit Triplex kit (Genes2Life, Irapuato, Guanajuato, Mexico). If a swab was not collected, the patient was serologically assayed for SARS-CoV-2 using the Panbio COVID-19 IgG/IgM rapid test (Abbott Laboratories, Chicago, IL).- Serologic tests were performed at the participating sites and qRT-PCRs were performed at the Laboratorio MicroTec, a reference laboratory in Mexico City. Swabs were not taken from patients who considered the qRT-PCR testing to be cost-prohibitive. Patients with positive qRT-PCR results had confirmed acute SARS-CoV-2 infections. Patients that contained SARS-CoV-2 IgM, either in the presence or absence of SARS-CoV-2 IgG, were considered to have probable acute or recent SARS-CoV-2 infections. Patients with SARS-CoV-2 IgG in the absence of IgM had probable past SARS-CoV-2 infections.

An aliquot of each serum was transported to Iowa State University and serologically and antigenically tested for seven arboviruses known to occur in Guerrero (CHIKV, DENV1–4, WNV, and ZIKV).^3^^–^^5^ To identify patients with acute CHIKV infections, sera were assayed for CHIKV IgM using the CHIKjj Detect IgM ELISA Kit (InBios International Inc., Seattle, WA). The laboratory criteria for the diagnosis of chikungunya, as established by the WHO, is the isolation of CHIKV from acute serum, detection of CHIKV IgM or RNA in acute serum or a 4-fold increase in CHIKV IgG titer in sera collected at least 3 weeks apart.^9^ A patient has confirmed chikungunya if at least one of the aforementioned laboratory tests yields a positive result irrespective of the clinical presentation. Therefore, all patients with CHIKV IgM met the case definition for chikungunya.

All sera with CHIKV IgM were further assayed by RT-PCR. Complementary DNAs were generated using Superscript III reverse transcriptase (ThermoFisher, Waltham, MA), and PCRs were performed using high-fidelity *Taq* polymerase (ThermoFisher) in accordance to the manufacturer’s instructions. Primers specific to a 445-nt region of the CHIKV E1 gene were used (forward primer: 5′- GTACAGCAGAGTGTAAGGA-3′, reverse primer: 5′- TCTTCGCTCTCAGGCGTG-3′'). RT-PCR products were purified using the purelink gel extraction kit (ThermoFisher) and sequenced using a 3730 × 1 DNA sequencer (Applied Biosystems, Foster City, CA). All sera with CHIKV IgM were also assayed by plaque reduction neutralization test (PRNT) using an isolate of CHIKV (strain CH-R-1950) originally recovered from a patient in Tamaulipas, Mexico, in 2015.^6^ PRNTs were performed using African green monkey kidney (Vero) cells as previously described.^7^ Sera were tested at a starting dilution of 1:20, and titers were expressed as the reciprocal of highest serum dilutions yielding > 90% reduction in the number of plaques (PRNT_90_).

To identify patients with acute flavivirus infections, sera were assayed using the Human Dengue Virus NS1 Antigen ELISA Kit (MyBioSource Inc., San Diego, CA). The ELISA is not DENV-specific because the flavivirus nonstructural protein 1 contains group-reactive epitopes.^8^ If flavivirus antigen was detected, the patient was considered to have an acute flavivirus infection. All antigen-positive sera were titrated and tested by PRNT to identify the flavivirus(es) responsible for the infections. PRNTs were performed using DENV-1 (strain Hawaii), DENV-2 (strain NGC), DENV-3 (strain H-87), DENV-4 (strain 241), WNV (strain NY99-35261-11), and ZIKV (strain PRVABC59). Viruses were obtained from the WHO Center for Arbovirus Reference and Research, which is maintained at the Division of Vector-Borne Infectious Diseases, Centers for Disease Control and Prevention in Fort Collins, CO. For etiologic diagnosis, the PRNT_90_ antibody titer to the respective virus needed to be at least 4-fold greater than that to the other flaviviruses tested.

The ages of the patients in the study population ranged from 4 to 89 years, with mean of 45.7 years. There were 87 females and 89 males. Half of the patients presented at the LLA (88 patients) and the remainder at the HGAPTA and LACAC (55 and 33 patients, respectively). Twenty (11.4%) patients had laboratory-confirmed acute SARS-CoV-2 infections, 96 (54.5%) patients had probable acute or recent SARS-CoV-2 infections and 60 (34.1%) patients had probable past SARS-CoV-2 infections.

Eighteen patients with evidence of SARS-CoV-2 infection contained CHIKV IgM in their sera (Table 1). These patients had CHIKV PRNT_90_ titers ranging from 20 to 1280. Six of these patients also contained CHIKV RNA (Genbank Accession Nos. OL440054-OL440059). The number of CHIKV RNA-positive patients is likely an underestimate because the sera were transported to Iowa State University on ice packs instead of dry ice, which is not sold in Guerrero. Of the 18 CHIKV IgM-positive patients, two patients had confirmed acute SARS-CoV-2 infections, seven had probable acute or recent SARS-CoV-2 infections, and nine had probable past SARS-CoV-2 infections. The two CHIKV IgM-positive patients with confirmed acute SARS-CoV-2 infections were negative for CHIKV RNA. One patient was a 24-year-old man who developed symptoms in May 2020. The other patient was a 62-year-old man with illness onset in July 2020.

**Table 1:** Patients with concurrent and close sequential SARS-CoV-2 and chikungunya virus infections, Guerrero, 2020–2021

Patient ID	Illness onset (month/year)	Performance site	Gender	Age (years)	SARS-CoV-2 diagnostic assay			CHIKV IgM ELISA	CHIKV RT-PCR	CHIKV PRNT_90_ titer
					RT-PCR	IgM test	IgG test			
HG122	05/2020	HGAPTA	M	24	+	NT	NT	+	–	40
HG127	05/2020	HGAPTA	F	27	NT	+	–	+	+	20
LL005	07/2020	LLA	M	62	+	NT	NT	+	–	640
LL017	10/2020	LLA	M	24	NT	–	+	+	–	640
LL018	10/2020	LLA	F	57	NT	–	+	+	–	160
LL042	11/2020	LLA	F	44	NT	+	+	+	–	160
LA100	12/2020	LACAC	M	38	NT	–	+	+	+	20
LA101	12/2020	LACAC	M	52	NT	–	+	+	–	320
LL057	01/2021	LLA	M	74	NT	+	+	+	+	20
LL058	01/2021	LLA	M	74	NT	+	+	+	+	1,280
LL059	01/2021	LLA	M	80	NT	–	+	+	+	20
HG172	02/2021	HGAPTA	F	10	NT	–	+	+	–	40
LA120	02/2021	LACAC	M	30	NT	+	+	+	–	640
LL062	02/2021	LLA	M	31	NT	+	+	+	+	320
LL064	02/2021	LLA	F	43	NT	–	+	+	–	20
LL070	02/2021	LLA	F	26	NT	–	+	+	–	160
LL078	03/2021	LLA	M	44	NT	+	+	+	–	640
LL084	03/2021	LLA	M	27	NT	–	+	+	–	160

+ = positive; – = negative; CHIKV = chikungunya virus; F = female; HGAPTA = Hospital General Adolfo Prieto in Taxco de Alarcón; LACAC = Laboratorio de Análisis Clínicos Avellaneda in Chilpancingo; LLA = Labymedic Laboratorios in Acapulco; M = male; NT = not tested; PRNT = plaque reduction neutralization test; RT-PCR = reverse transcriptase polymerase chain reaction; SARS-CoV-2 = severe acute respiratory syndrome coronavirus 2.

Sera from 16 patients contained flavivirus antigen (Table 2). Of these, one patient had a confirmed acute SARS-CoV-2 infection, 10 had probable acute or recent SARS-CoV-2 infections, and five had probable past SARS-CoV-2 infections. The flaviviruses responsible for the infections were DENV1 (two patients), DENV2 (five patients), DENV3 (three patients), ZIKV (three patients), and an undetermined flavivirus (three patients). The patient that contained both SARS-CoV-2 RNA and flavivirus antigen was seropositive for DENV1. The patient was a 40-year-old woman who developed symptoms in June 2020. Of the 10 flavivirus antigen-positive patients with probable acute or recent SARS-CoV-2 infections, one patient was seropositive for DENV1, three were seropositive for DENV2, two were seropositive for DENV3, two were seropositive for ZIKV, and two had antibodies to an undetermined flavivirus.

**Table 2:** Patients with concurrent and close sequential SARS-CoV-2 and flavivirus infections, Guerrero, 2020–2021

Patient ID	Illness onset (month/year)	Performance site	Gender	Age (years)	SARS-CoV-2 diagnostic assay			Flavivirus NS1 ELISA	PRNT outcome
					RT-PCR	IgM test	IgG test		
HG004	05/2020	HGAPTA	M	68	NT	+	+	+	DENV3
HG005	05/2020	HGAPTA	F	37	NT	–	+	+	DENV3
HG011	05/2020	HGAPTA	M	27	NT	+	–	+	DENV1
HG012	06/2020	HGAPTA	F	40	+	NT	NT	+	DENV1
HG013	06/2020	HGAPTA	F	28	NT	–	+	+	FLAVI
HG017	06/2020	HGAPTA	M	35	NT	+	–	+	DENV3
LL001	07/2020	LLA	M	66	NT	+	+	+	DENV2
LL006	07/2020	LLA	M	62	NT	–	+	+	DENV2
LL007	07/2020	LLA	M	61	NT	–	+	+	ZIKV
LL012	08/2020	LLA	F	52	NT	–	+	+	DENV2
LL034	11/2020	LLA	F	54	NT	+	+	+	DENV2
LL041	11/2020	LLA	F	45	NT	+	+	+	ZIKV
LL048	11/2020	LLA	F	44	NT	+	+	+	FLAVI
LL054	01/2021	LLA	M	17	NT	+	–	+	DENV2
LA016	01/2021	LACAC	M	36	NT	+	+	+	FLAVI
LL080	03/2021	LLA	M	72	NT	+	+	+	ZIKV

+ = positive; – = negative; DENV1 = dengue virus 1; DENV2 = dengue virus 2; DENV3 = dengue virus 3; F = female; FLAVI = undetermined flavivirus; HGAPTA = Hospital General Adolfo Prieto in Taxco de Alarcón; LACAC = Laboratorio de Análisis Clínicos Avellaneda in Chilpancingo; LLA = Labymedic Laboratorios in Acapulco; M = male; NT = not tested; PRNT = plaque reduction neutralization test; RT-PCR = reverse transcriptase polymerase chain reaction; SARS-CoV-2 = severe acute respiratory syndrome coronavirus 2; ZIKV = Zika virus.

Our data indicate that a subset of patients had concurrent or close sequential infections between SARS-CoV-2 and various arboviruses—namely CHIKV, DENV1, DENV2, DENV3, and ZIKV. Other studies have reported patients with concurrent SARS-CoV-2 and DENV infections.^9^^–^^17^ Most patients had DENV1 infections, but others had DENV2 or DENV3 infections. To the best of our knowledge, coinfections or close sequential infections between SARS-CoV-2 and CHIKV or ZIKV have not been reported. None of the patients with acute SARS-CoV-2 infections in Angola in 2021 had evidence of CHIKV or ZIKV infection.^16^ Coinfections were not reported during the concurrent outbreaks of SARS-CoV-2, CHIKV, DENV, and ZIKV in Espírito Santo State, Brazil, in 2020.^18^

In conclusion, we report apparent concurrent and close sequential infections between SARS-CoV-2 and select arboviruses in Guerrero, Mexico. SARS-CoV-2 and the arboviruses under investigation produce overlapping clinical manifestations (i.e., fever, headache, fatigue, and myalgia), complicating the diagnosis of coinfections.^19^^,^^20^ There is also considerable overlap in the laboratory characteristics associated with SARS-CoV-2 and DENV infections (i.e., thrombocytopenia, lymphopenia, leukopenia, and elevated liver enzymes).^20^ Failure to identify coinfections can adversely affect patient outcomes due to delays in the implementation of disease-specific treatments, such as the isolation of COVID-19 patients and the venous hydration of dengue patients. Our findings underscore the important need to perform differential diagnosis in regions where these viruses cocirculate. Prospective epidemiological studies are needed to determine whether SARS-CoV-2 potentiates infections with arboviruses or vice versa.
